# Abstract withdrawn

**DOI:** 10.1186/1742-4690-9-S1-P25

**Published:** 2012-05-25

## Abstract withdrawn

